# Effect of Phase Shift from Corals to Zoantharia on Reef Fish Assemblages

**DOI:** 10.1371/journal.pone.0116944

**Published:** 2015-01-28

**Authors:** Igor C. S. Cruz, Miguel Loiola, Tiago Albuquerque, Rodrigo Reis, José de Anchieta C. C. Nunes, James D. Reimer, Masaru Mizuyama, Ruy K. P. Kikuchi, Joel C. Creed

**Affiliations:** 1 Programa de Pós Graduação em Ecologia, Universidade do Estado do Rio de Janeiro, Rio de Janeiro, RJ, Brazil; 2 Laboratório Recifes de Corais e Mudanças Globais, Instituto de Geociências, Universidade Federal da Bahia, Salvador, BA, Brazil; 3 Programa de Pós Graduação em Ecologia e Biomonitoramento, Universidade Federal da Bahia, Salvador, BA, Brazil; 4 Laboratório de Ecologia Bentônica, Instituto de Biologia, Universidade Federal da Bahia, Salvador, Bahia, Brazil; 5 Molecular Invertebrate Systematics and Ecology Laboratory, Graduate School of Engineering and Science, University of the Ryukyus, Nishihara, Okinawa, Japan; 6 Laboratório de Ecologia Marinha Bêntica, Departamento de Ecologia, Instituto de Biologia Roberto Alcântara Gomes, Universidade do Estado do Rio de Janeiro—UERJ, PHLC Sala 220, Rio de Janeiro, RJ, Brazil; National University of Singapore, SINGAPORE

## Abstract

Consequences of reef phase shifts on fish communities remain poorly understood. Studies on the causes, effects and consequences of phase shifts on reef fish communities have only been considered for coral-to-macroalgae shifts. Therefore, there is a large information gap regarding the consequences of novel phase shifts and how these kinds of phase shifts impact on fish assemblages. This study aimed to compare the fish assemblages on reefs under normal conditions (relatively high cover of corals) to those which have shifted to a dominance of the zoantharian *Palythoa* cf. *variabilis* on coral reefs in Todos os Santos Bay (TSB), Brazilian eastern coast. We examined eight reefs, where we estimated cover of corals and *P.* cf. *variabilis* and coral reef fish richness, abundance and body size. Fish richness differed significantly between normal reefs (48 species) and phase-shift reefs (38 species), a 20% reduction in species. However there was no difference in fish abundance between normal and phase shift reefs. One fish species, *Chaetodon striatus*, was significantly less abundant on normal reefs. The differences in fish assemblages between different reef phases was due to differences in trophic groups of fish; on normal reefs carnivorous fishes were more abundant, while on phase shift reefs mobile invertivores dominated.

## Introduction

Phase shifts are one of the most drastic consequences of coral reef degradation [1–4]. This phenomenon is characterized by an abrupt decrease in coral abundance or cover and concurrent increase to dominance of non-reef-building organisms, such as algae and soft corals [5]. The consequence of this phenomenon is the loss of some ecosystem services, such as fishing and tourism [3], and changes in local biodiversity [1]. It has been estimated that 19% of coral reefs worldwide have been lost and another 35% are threatened [6], which makes this scenario alarming; it has been termed the "Coral Reefs Crisis" [3,4].

Despite the severity of this problem, to date the only mechanism extensively studied is the shift to dominance of macroalgae, leaving a large gap in our knowledge about the processes of phase shifts involving dominance to other types of organisms [7]. The relationship between change in fish assemblages and macroalgal dominance is well known [3,8–10], and although there is no consensus [11–13] the loss of herbivorous fish through overfishing is considered one of the causes of dominance by macroalgae [14–16]. There is also a large information gap regarding the consequences or relationships between these other kinds of benthic phase shifts and fish assemblages.

The effects of composition and structure of benthic assemblages and associated benthic composition, and environmental structural complexity on reef fish assemblages, have been well documented since the beginning of the 1970s [17–24]. A shift in benthic structure can drive a change in abundance and composition of fish species that are directly linked to specific benthic trophic groups. This change can affect those species which were previously abundant and/or those that had key ecological roles in the community, such as herbivores, which control the algae that compete with framework building organisms [3,8,9,16]. The loss of key species implies alterations in the community structure as well as in ecosystem stability [10,15,25]. However, the feedback by which fishes and benthic assemblages influence each other in coral reef ecosystems remains poorly understood. Understanding these ecological interactions is essential in order to prevent and/or manage phase shift situations [26,27].

The present study therefore aims to compare fish assemblages on normal condition reefs, with relatively high coverage of corals, to those shifted to a dominance of the zoantharian *Palythoa* cf. *variabilis* (initially identified as *Epizoanthus gabrieli* [28]) in Todos os Santos Bay (TSB), Brazilian eastern coast. To investigate the differences between these two reef conditions we tested if fish assemblages on phase shift reefs had: (i) less species; (ii) lower abundance; (iii) different trophic structures; and (iv) reduced fitness [29], by perturbing the cleaning service supplied by the Barber (Neon) Goby *Elacatinus figaro*. We also investigated whether a reduced abundance of a zoantharian predator, the butterfly fish *Chaetodon striatus*, might be responsible for the zoantharian outbreak (parallel to the loss of herbivores in coral-to-algae reef shifts).

## Materials and Methods

### Study area

**Fig 1:**
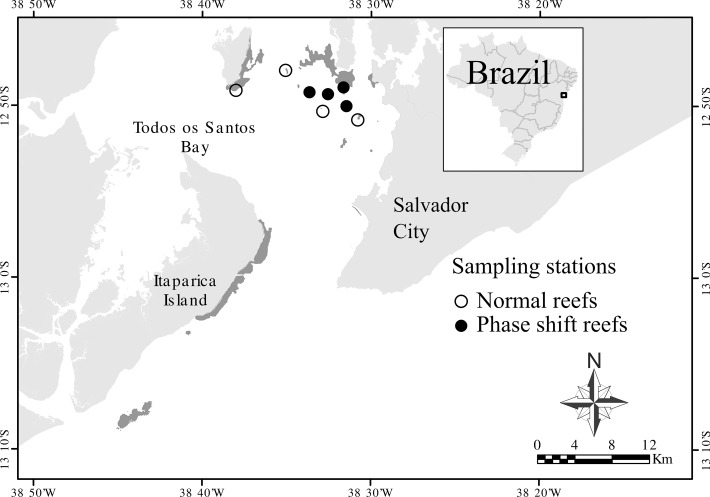
Sampling stations in Todos os Santos Bay. Reefs under normal conditions are represented by white circles while reefs under phase shift (high zoantharian cover) are signed by black circles.

This study was carried out in Todos os Santos Bay (TSB) (12°50'S and 38°38'W). It is the second largest embayment in Brazil (about 1235 km^2^), located on the Brazilian eastern coast, and surrounded by Salvador City, the third largest urban area in the country [30] (Fig. 1). The Brazilian eastern coast has the highest coral diversity in the South Atlantic Ocean [31,32]. The TSB is an environmentally protected area (EPA), equivalent to the landscape/seascape IUCN category [33]. Nevertheless, this bay receives numerous environmental stressors such as sewage, industrial runoff, overfishing, dynamite fishing, disorderly occupation of the coastline and has suffered environmental disasters such as contamination by lead, mercury, and oils spills [34]. Faced with these stressors, identifying the probable cause of this *Palythoa* phase shift is not easy, although Yang et al. [35], performing a study in Okinawa, Japan, pointed out that the dominance of *P*. *tuberculosa* in some reef areas may be related to nutrient input from terrestrially derived river run-off. Costa et al. [36], in their review article of the role of nutrient overloading on Brazilian coral reefs, also mention how *Palythoa* benefits from nitrification enrichment in coastal reefs. Despite heavy impacts some reefs in TSB still have a coral cover considered high for the region (between 8% and 27% *c*.*f*. Cruz et al. [36]) relative to other Brazilian inshore reefs (3.6% *c*.*f*. Leão et al. and Costa et al. [37,38]). On the other hand some reefs (total area of approximately 10.5 km^2^) are dominated by the zoantharian *Palythoa* cf. *variabilis* and have a coral cover lower than 3.6% [28].

### Ethics statement

We used a non-destructive methodology to assess fish assemblages. These data were collected using visual census and recorded images. This bay is an Environmentally Protected Area, and according to Brazilian law SNUC (National System of Conservation Areas) [39] this kind of conservation unit does not require a license for studies that use non-destructive methods. Only *Palythoa* specimens as detailed below were collected under license N° 24958–1 issued by the Brazilian Environment Ministry and sent to Japan for genetic analysis under transport and genetic heritage license N° 14BR013578/DF, also issued by the Brazilian Environment Ministry.

### Analyses of *Palythoa* specimens

*In situ* images were collected of each *Palythoa* (Anthozoa: Hexacorallia: Zoantharia: Sphenopidae) specimen before collection. Four *Palythoa* specimens were collected from the study site on a phase shift reef by hand and preserved in 95% ethanol. Only small fragments consisting of 3 polyps were collected from each sampled *Palythoa* colony. The specimens were collected from Poste 1 (12°49'20.1"S 38°33'35.4"W) within the study site. From *in situ* observations and digital images, the *Palythoa* specimens were believed to belong to a single species, and all specimens were zooxanthellate, with heavily encrusted body walls, and polyps were separated from each other and only joined by a thin coenenchyme.

Molecular analyses were performed on four *Palythoa* specimens (specimen numbers 418–421). DNA extraction with a DNeasy Blood and Tissue Kit (Qiagen, Tokyo, Japan) followed the manufacturer’s instructions. Sequences of mitochondrial 16S ribosomal DNA (mt 16S rDNA) and the internal transcribed spacer region of ribosomal DNA (ITS-rDNA) were amplified using previously reported methods and primers [40,41]. Amplified PCR products were visualized on 1.0% agarose gel and cleaned up following a shrimp-alkaline phosphatase treatment. Subsequently, mt 16S rDNA and ITS-rDNA products were sequenced at Fasmac Co., Ltd (Kanagawa, Japan).

Newly acquired mt 16S rDNA and ITS-rDNA sequences were deposited in GenBank (GenBank Accession Numbers KP174720-KP174725). Following the methodology of Bo et al. [42], we compared our newly acquired sequences for similarity with previously reported Zoantharia sequences by: 1) National Center for Biotechnology Information’s Basic Local Alignment Search Tool (NCBI BLAST) [43], and 2) by manual visual comparison using alignment software Se-Al v2.0a11 (University of Edinburgh). As in Bo et al. [42], our newly acquired sequences were compared by similarity only with no additional phylogenetic analyses, as previous research has shown that mt 16S rDNA and ITS-rDNA sequences combined are generally accurate in identifying zoantharian specimens to the species level [44,45].

### Data collection

In 2011 we sampled eight reefs where we collected data on reef fish assemblages and benthic cover of the corals and *P*. cf. *variabilis*. We used a video transect method to assess benthic assemblages [46,47]. We performed six parallel band transects at each sampled station, registered on digital video. We used the video-transect method with a 40 cm long aluminum rod coupled to the filming system to standardized the image area [48]. At this distance the transect width sampled by the camera was 0.2 m. The length of the belt-transects was 20 m, which gave a sampled area of 24 m^2^ per station. The relative coverage of corals and *P*. cf. *variabilis* on each sampled reef was estimated using the software CPCe 3.6 [49]. We divided the belt transects into successive frames, on which 20 randomized points were placed to estimate cover of the benthic organisms. The stations were divided in phase-shift reefs and normal reefs: reefs presenting a zoantharian coverage greater than 30% and coral cover lower of 3.6% were considered to have shifted phase (12°49'45"S 38°32'52"W; 12°49'31"S 38°32'08"W; 12°49'20"S 38°33'36"W; 12°50'00"S 38°31'31"W) while those on which zoantharian coverage did not exceed 10% were considered ‘normal’ (12°48'33"S 38°37'35"W; 12°50'43"S 38°30'47"W; 12°50'13"S 38°32'57"W; 12°47'54"S 38°35'02"W).

We sampled fish assemblages with visual censuses. In each station, we used an adaptation of Atlantic and Gulf Rapid Reef Assessment (AGRRA) Fish Protocol [50] with 10 band transects of 30 meters long by 2 meters wide, covering a total sampled area of 600 m^2^. In each transect we estimated the abundance of all observed species during 15 minutes. The fish species found were classified into the following trophic groups: carnivores, mobile invertivores, sessile invertivores, piscivorous, planktivores, omnivores, territorial herbivores, roving herbivores and cleaners as according to Ferreira et al. and Medeiros et al. [51–53].

### Data analysis

The species richness was estimated and compared by a rarefaction analysis using an accumulated species curve *Mao tau* (Sobs) and their respective confidence interval at 95% [54,55]. Each transect was used as samples totalled 40 samples on normal reefs and another 40 in phase shift reefs. Each set of 40 samples was distributed 999 times in random order to calculate average species accumulation (Sobs) and its confidence interval [56]. The confidence interval was calculated using Student’s *t* distribution: if the limits did not overlap the data sets were judged as different [57,58]. In addition, qualitative ACE, Chao 1, Jack 1 and quantitative ICE, Chao 2 and Jack 2 indices of species number were used to estimate the richness of fishes in phase shift reefs compared with species accumulation on normal reefs. We performed these analyses using EstimateS 8.2.0 software [59]. We tested the difference in the abundance of fishes, density of *Chaetodon striatus* and *Elacatinus figaro* between normal and phase shift reefs with Student’s *t*-test [57] using StatSoft STATISTICA, version 8.0 software.

Standard multivariate analyses were performed to explore differences in trophic guilds between normal and phase shift reefs (*α* = 0.05), a multivariate nonparametric test based on the Bray-Curtis similarity index [60,61]. The similarity percentage (SIMPER) routine was used to identify which trophic guilds were important in the groupings identified by ANOSIM [60–62]. Finally, multi-dimensional scaling (MDS) was applied and the Bray-Curtis similarity index was used to illustrate patterns of similarities and differences [60,61]. Theses multivariate analyses were undertaken using PRIMER 6 (Primer-E) software.

## Results

### *Palythoa* specimen identification

Newly acquired sequences of mt 16S rDNA and ITS-rDNA from specimens matched closely with previously reported *Palythoa* species’ sequences.

For mt 16S rDNA, sequences were obtained from all four examined specimens, and were identical over their entire length (480 base pairs). By BLAST comparison, these sequences were identical to previously reported sequences from *Palythoa heliodiscus* (GenBank Accession Number AB219224) and *Palythoa* cf. *heliodiscus* (HM754466), etc.) from the Indo-Pacific and the aquarium trade, respectively.

For ITS-rDNA, only short sequences were obtained from two examined specimens (418, 419), and these were identical to each over their entire length (178 base pairs). By BLAST comparison, these sequences were most similar (177/178 base pairs) to a previously reported sequence from *Palythoa* aff. *variabilis* (JX119123) from Florida.

Recent research has shown that there are closely related sibling species of *Palythoa* in the Atlantic and Indo-Pacific Oceans. Among these are the species *P*. *heliodiscus* (Indo-Pacific) and *P*. *variabilis* (Atlantic), which have identical mt 16S rDNA and similar ITS-rDNA sequences [63]. Therefore, for this study, based on mt 16S rDNA and ITS-rDNA sequence similarity, specimen sampling location, and general morphological characteristics, we identified the specimens from TSB as *Palythoa* cf. *variabilis*.

### Community assessment

In normal reefs the coral coverages were 16.1% ±6.4 (SD), 34.2% ±3.82, 19.1% ±2.4 and 23.7% ±3.8 while in phase shifted reefs coverages were 2.4% ± 2.0, 2.0% ± 0.7, 0.5% ± 0.4 and 0.9% ± 0.6. *Palythoa* cf. *variabilis* coverages were 0.1% ± 0.1, 0.1% ± 0.1, 0% ± 0 and 8.4% ± 13.7 on normal reefs and 88.6% ± 6.2, 60.3 ± 10.7%, 33.8% ± 10.2 and 61.5% ± 4.4 on phase shift reefs.

**Fig 2:**
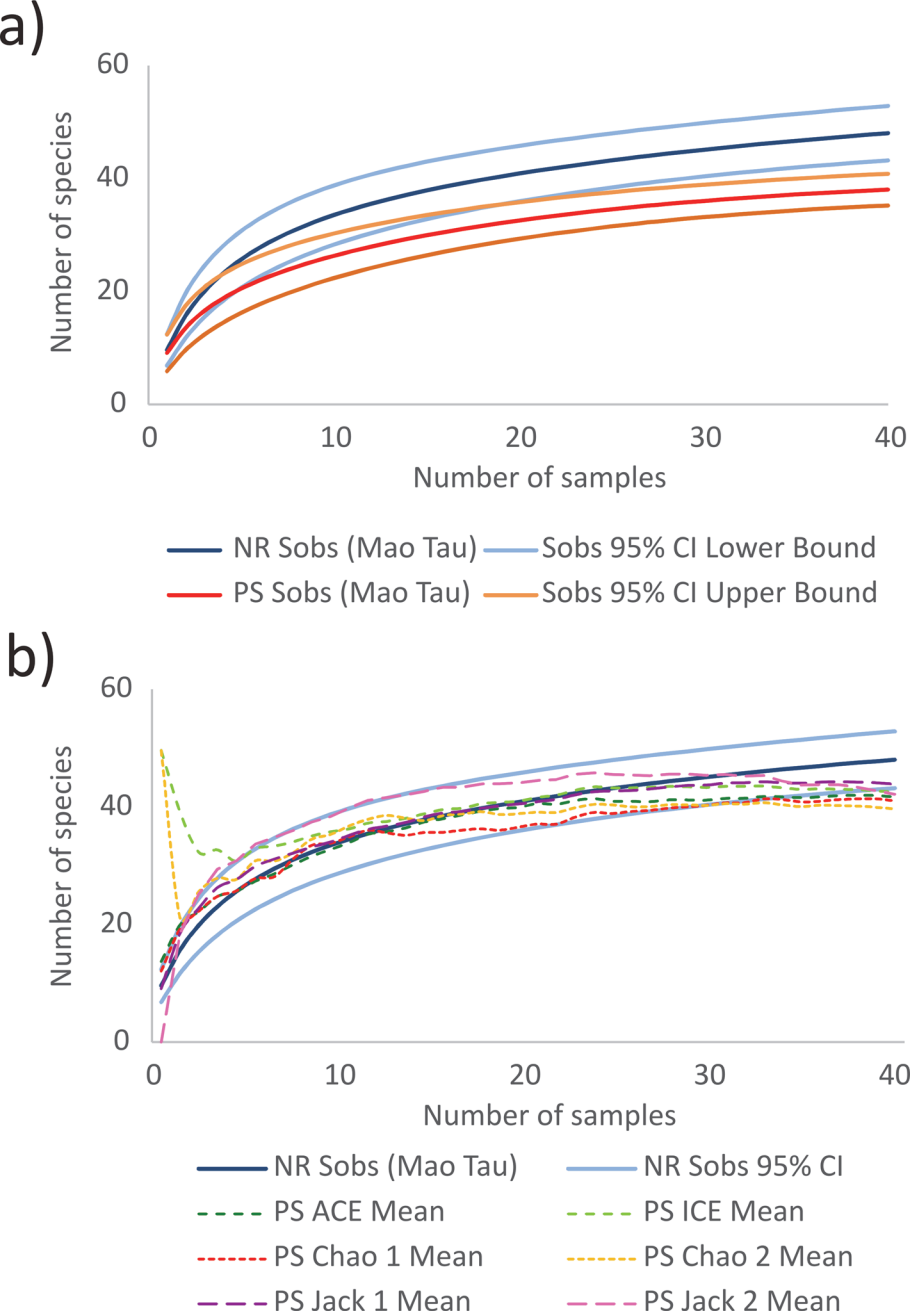
Probabilities of numbers of fishes species by number of samples with (a) number of species observed (Sobs) in normal (NR) and phase shift (PS) reefs with their respective confidence intervals (95%), and (b) estimators of species richness of reefs in phase shift (PS) in comparison with the number of species observed in normal reef (NR) and its confidence interval less than 95%.

We found a significant difference in the fish richness between phase-shift reefs with 38 species and normal reefs with 48 species (Fig. 2A). In addition, all richness estimators of phase-shift reefs showed a lower number of species than the number of species observed on normal reefs (Fig. 2B). Furthermore, the only richness estimator that still overlapping the confidence interval of normal reefs Sobs was Jack 1. Moreover, the normal reefs had higher diversity than phase shift reefs (*H’* = 2.74 ± 0.01(SD), *J’* = 8.61 ± 0.01 and *H’* = 2.11 ± 0.01; *J’* = 5.03 ±0.01, respectively).

**Fig 3:**
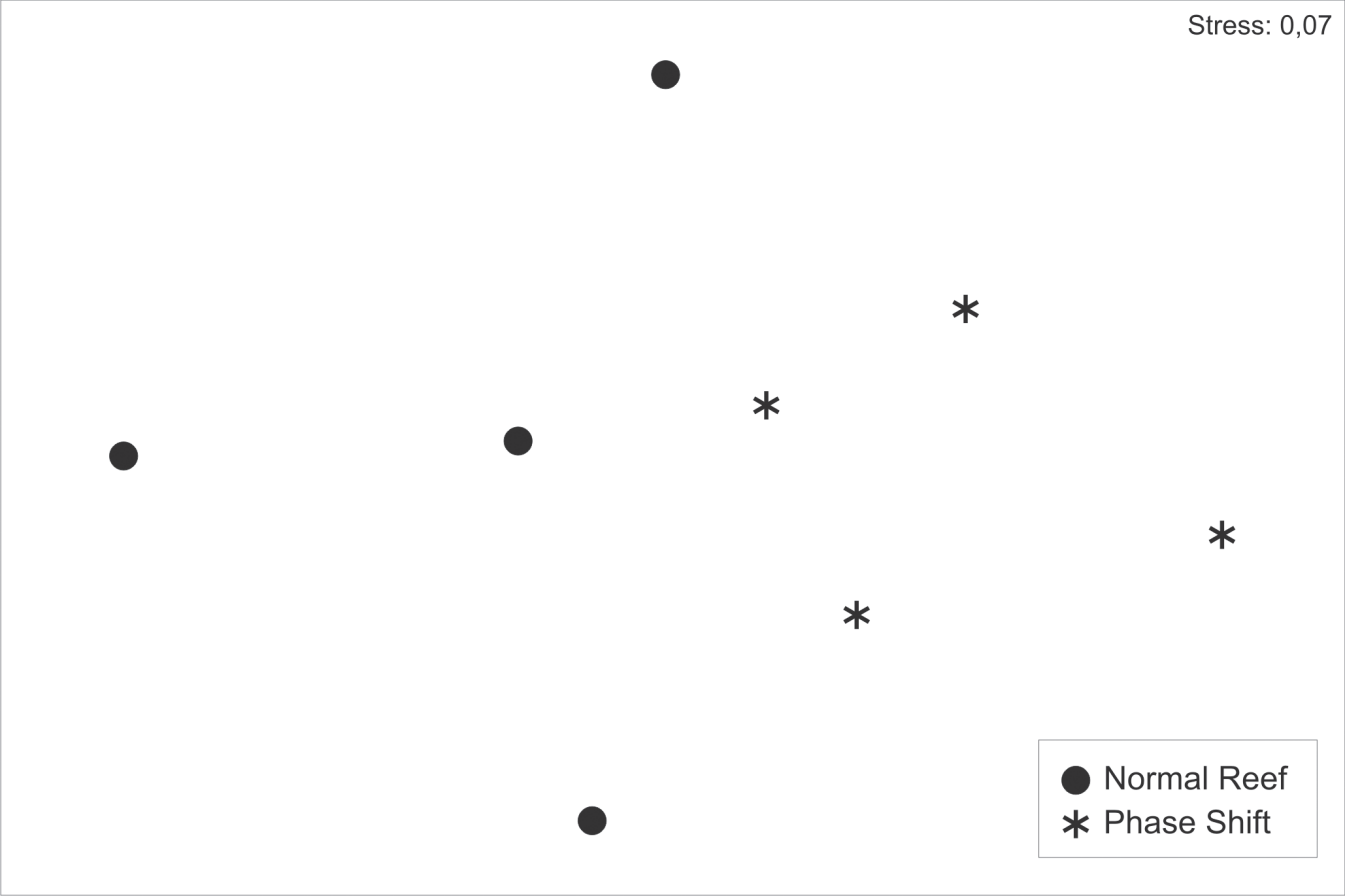
Multidimensional scaling of trophic structure of reef fishes assemblage on normal reefs (represented by dark balls) and phase shift reefs (dominated by the zoantharian *Palythoa* cf. *variabilis*), which in figure are represented by asterisks.

The analysis of similarity (ANOSIM) showed a difference in patterns of dominance in fish assemblages, for trophic groups, between reef types (Global *R* = 0.479; *p* = 0.029). The percentage of dissimilarity (SIMPER) between these two groups was 41.4%, explained mainly (50%) by a higher number of mobile invertivores in phase shift reefs (Table 1). Moreover the sessile invertivores and carnivores were more abundant on unaffected reefs and together with the mobile invertivores represented 88.2% of the difference between the reef types. The difference between phases shift of reefs in relation to trophic groups was clear on the MDS (Fig. 3), with the total separation of normal and phase shift reef groups along the first axis.

**Table 1:** Dissimilarity between the trophic structure of reef fish on normal and phase shift (high zoantharian cover) reefs calculated by Similarity Analysis.

Average dissimilarity = 41.39	Normal reefs	Phase shift				
Species	AvDens.	Av.Dens.	Av.Diss	Diss/SD	Contrib%	Cum.%
Mobile invertivores	81.5	322.75	22.2	2.24	53.63	53.63
Sessile invertivores	166	107.25	8.22	1.2	19.86	73.49
Carnivores	133.25	105.25	6.09	1.32	14.72	88.21
Territorial herbivores	25.5	32.25	2.08	1.25	5.03	93.24

AvDens – Average of Density, AvDiss – Average of Dissimilarity, Diss/SD – Dissimilarity Standard Deviation, Contrib% – Percentage of Contribution to Dissimilarity, Cum.% – Cumulative Percentage Contribution to Dissimilarity.

**Fig 4:**
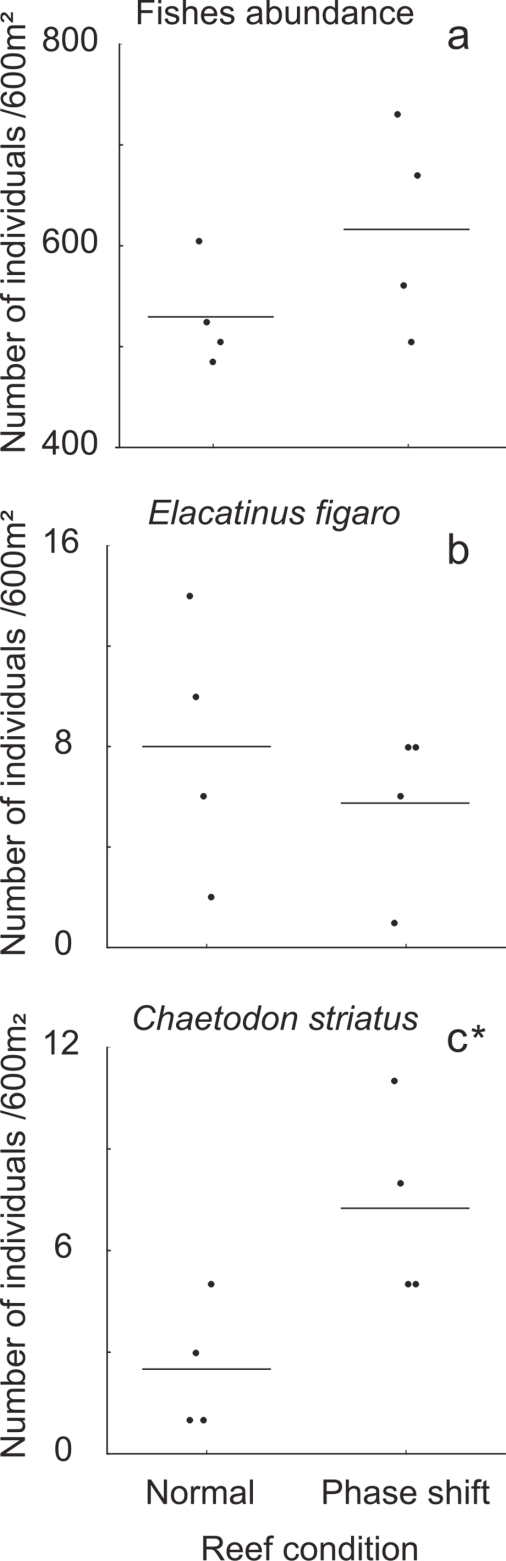
Density of fish per reef, where a point represents each station-value. The mean value for each group of reef is represented by the dash line and asterisk (*) marks the graph that had a significant difference (*p* < 0.05) according the Student's *t* test.

We observed that there was no difference in overall fish abundance between normal and phase shift reefs (Student’s *t*-Test, *t* = 1.555; *df* = 6; *p* = 0.173, Fig. 4A). In relation to *Elacatinus figaro* there was no significant difference between densities on the two groups of reefs (*t* = -0.734, *df* = 6, *p* = 0.490; Fig. 4B). Finally, normal reefs had significantly lower density of *Chaetodon striatus* than zoantharian dominated reefs (*t* = 2.752; *df* = 6; *p* = 0.033; Fig. 4C).

## Discussion

This is the first study to describe differences in fish assemblages associated with reefs under normal conditions (e.g. with satisfactory coral coverage) and reefs that became dominated by a fast-growing zoantharian and are now regime shifted. Despite the fact that fish richness sampled in both reef groups was relatively low (50 fish species of the 405 species registered in Brazilian reef habitats to date [64], the pattern is consistent with the number of species seen on other Brazilian reefs: 54 species reported by Chaves et al in Porto Seguro [65], 66 species reported by Kajewski and Floeter in Fernando de Noronha Archipelago [66] and 26 species reported by Medeiros et al in Picãozinho reef, located at northeaster of Brazilian coast [53]). The small number of individuals with sizes above 20 cm confirmed that the reefs of the TSB are not pristine and that these communities suffer the direct effects of human stressors as described by Dutra and Haworth [34], such as overfishing, destructive fishing, introduction of alien species, domestic and industrial sewage and oil spills. However, even in a community already under stress, the effects of phase shift on fish assemblages were evident. Under this scenario extra conservation attention needs to be given to these disturbed ecosystems in order to avoid a further worsening in reef degradation and consequent further loss of ecosystem goods and services.

We found that richness and diversity of fishes were lower on TSB phase shift reefs. This result may reflect a homogenization of the reefs under the phase shift situation. *Palythoa* cf. *variabilis* polyps are up to 4 cm long (Cruz, ICS pers. obs.) and when this species grows over the reef it obstructs and fills in reef shelters, substantially reducing rugosity by blanketing and smoothing the entire surface of the reef substratum. Moreover, the reduction of stony coral cover negatively modifies the balance between bioconstruction and bioerosion, reducing reef structural complexity [67–69]. The rugosity of a consolidated substrate is the ratio of a surface area with its planar projection and is directly proportional to the number of available shelters [24,70]. In coral reefs, the rugosity is directly proportional to the number and size of coral colonies, which in precipitating calcium carbonate, increase the structural complexity of habitats [71]. According to MacArthur and MacArthur and Tews et al. [72,73] this complexity influences the associated biodiversity and, consequently, the ecosystem function. Thus, we believe that the reduction of rugosity (i.e. habitat complexity) from increased *P*. cf. *variabilis* coverage is the main mechanism for observed changes in the local fish richness and diversity.

Positive correlation between structural complexity and reef fish diversity has been widely reported [17,18,21,22,24,74–77]. Additionally, Syms and Jones, Almany and Willis et al. [78–80] have confirmed that increasingly complex habitat provides more shelters against predators and facilitates coexistence, even for competitors, providing niche partitioning. Habitat complexity can therefore be an important factor, explaining the richness and diversity of species and potentially changing competitive interactions and survival [78,81].

Another possible explanation for this richness pattern is the decrease of heterogeneity, by the loss of substrate variety caused by the *P*. cf. *variabilis* dominance at phase shift reefs, and according to Tews et al. [73], this process is extremely dependent on scale. Heterogeneity loss probably more intensely affects small fishes with limited mobility. On the same scale, this phenomenon probably affects the mobile invertebrate assembly and may increase the abundance of some invertebrate groups to the detriment of others. Further support of this idea is shown by the fact that *Palythoa* species are considered to host many invertebrate species which find refuge among their soft polyps [82,83]. This could explain the abundance of mobile invertivores on phase shift reefs. Unlike sedentary fishes, mobile invertivore species can migrate to other reefs in order to find food resources.

In contrast with our results on subtropical Brazilian rocky shores Mendonça-Neto et al. [84] found that over patches with a dominance of the zoantharian *Palythoa caribaeorum* (~70% of benthic cover) the richness and abundance of fishes were higher than over patches with low zoantharian cover (~10%). According to Ferreira et al. [51] *P*. *caribaeorum* is found in shallower zones of these environments, which characteristically have crevices and are therefore considered to be areas of greater structural complexity. The fish richness found by Mendonça-Neto et al. [84] may in fact be associated with benthic complexity rather than being directly related with zoantharian coverage.

The two reef conditions investigated in this study did not differ significantly in total fish abundance. Other studies have indicated a weak relationship between fish abundance and complexity, in contrast to the clear negative pattern observed for richness. However, our results are in agreement with those of Risk, Gladfelter and Gladfelter, and Carpenter et al. [17,18,85], who also found no effect of complexity on species’ abundance.

On zoantharian dominated reefs we observed large number of the grunts *Haemulon aurolineatum* and *H*. *steindachneri* (S1 Table). Grunts are very abundant in shallow reef environments, and are nocturnal and mobile invertivores that forage mostly on zooplankton (basically juveniles) and/or macrobenthic invertebrates (adults) associated with soft substrates [51,86–88]. These two haemulids had an average length of 10 cm and were responsible for greater abundance of this size class on phase shift reefs (unpublished data collected following the AGRRA Fish Protocol [50]).

The trophic structure of fishes differed between reef groups and the large number of mobile invertivores explained this difference as they were favored by or at least uninfluenced by zoantharian dominance (S2 Table). Different from grunts, other fish groups show direct relationships with reef benthic communities, where they seek refuge against predators, find food, utilize cleaning stations, or perform reproduction and have spawning sites [9]. Sessile invertivores and carnivores are good models of groups that interact with the reef substratum. On normal reefs with heterogeneous substrate these two groups were more abundant. Roving herbivores were the other group that did not favor zoantharian dominance; Mendonça-Neto et al. [84]’s results further support the idea that roving herbivores tend to be abundant in zones without zoantharian mats. Despite this, other studies have found no relationship between the abundance of herbivorous fishes and algae cover [89,90]; the absence of zoantharians may provide more space for algal growth, which potentially benefits herbivores [84].

The corals *Montastraea cavernosa*, *Mussismilia hispida* and *Siderastrea* spp. are the most abundant reef builders on TSB coral reefs [36] and it is known that in the Caribbean the cleaner fish *Elacatinus pallens* and *E*. *pilepis* prefer microhabitats provided by *Montastraea* and *Siderastrea* [91]. The coverages on normal reef were 18.88% ±7.12 (SD), 0.98% ±0.80 and 1.88% ±0.80 respectively, while on phase shift reef they were 1.28% ±0.84, 0.01% ±0.01 and 0.02% ±0.02 and the density on normal reef were 3.26 /m^2^ ±0.99, 0.41 /m^2^ ±0.41 and 1.12 /m^2^ ±0.41 while on phase shift reef they were 0.39 /m^2^ ±0.27, 0.02 /m^2^ ±00.01 and 0.03 /m^2^ ±00.02. We therefore expected a greater abundance of the endemic (Brazil) Barber goby *E*. *figaro* on normal reefs of the TSB. However we did not observe higher abundance of this cleaner on normal reefs. On Brazilian reefs *E*. *figaro* is the main cleaner, being the only species that has an obligate cleaning behavior [92], and is strongly associated to the benthos through the corals that are used to demarcate its cleaning station [92]. The lack of a significant difference between the normal and phase shift reefs indicates the behavioral plasticity of this species, and demonstrates that it can also use other organisms such as crustose coralline algae, echinoderms or even zoantharians to mark its cleaning stations [92]. Another reason for the absence of difference in *E*. *figaro* abundance between the two sets of reefs may be the fishing pressure suffered by this species in the TSB. Due to its disruptive coloration *E*. *figaro* is a preferred target for the ornamental fish trade [93] and this activity, extensively practiced in the studied reefs, may reduce their natural stocks resulting in similar abundances.

We found a higher abundance of the butterfly fish *Chaetodon striatus* on phase shift reefs in TSB, a result contrary to what we expected. We can therefore reject the hypothesis that *C*. *striatus* is capable of exerting top-down control on *P*. cf. *variabilis*. According to Mendonça-Neto et al. [84] *Palythoa* patches provide optimal conditions for sessile invertebrate feeders (mainly chaetodontids) which may forage on polyps. Chaetodontids are highly associated with corals, especially Pacific species [18,20,94,95]. However, according to Bonaldo et al. [96], *C*. *striatus* forages on zoantharian colonies so the availability of food on phase shift reefs probably supported the higher density of butterfly fishes found on phase shift reefs. Studies evaluating what are the potential predators for *P*. cf. *variabilis* on Brazilian coral reefs are needed in order to understand underlying interactions.

In summary, we found that phase shift to *P*. cf. *variabilis* dominance had a negative effect on the diversity of fish fauna in TSB coral reefs. The impacted reefs showed (i) a lower number of fish species, and (ii) an altered trophic structure of fish assemblages, (iii) but without differences in fishes abundace. We rule out the possibility of (iv) top-down control of *P*. cf. *variabilis* from predation by *C*. *striatus* (yet also demonstrated that this fish species is favoured by the phase shift), and (v) did not see any effect of this phase shift on the ecosystem services provided by the cleaner *E*. *figaro* due to reduced coral cover. Further studies are needed to understand both how this phase shift alters the trophic structure of fish assemblages and the underlying ecological processes involved.

## Supporting Information

S1 Table Reef fishes species recorded in each station. (XLSX)

S2 Table Trophic guilds recorded in each station. (XLSX)
